# Revolutionizing Hyperlipidemia Treatment: Nanoencapsulated CoQ10 and Selenium Combat Simvastatin-Induced Myopathy and Insulin Resistance in Rats

**DOI:** 10.34172/apb.2024.010

**Published:** 2023-09-23

**Authors:** Hagar B. Abo-Zalam, Ezz El Deen El Denshary, Rania A. Abdalsalam, Islam A. Khalil, Mahmoud M. Khattab, Mohamed A. Hamzawy

**Affiliations:** ^1^Department of Pharmacology and Toxicology, Faculty of Pharmacy, 6th of October University, 6th of October, Giza, Egypt.; ^2^Department of Pharmacology and Toxicology, Faculty of Pharmacy, Cairo University, Cairo, Egypt.; ^3^School of Pharmacy, New Giza University, Giza, Egypt.; ^4^Department of Pharmaceutics and Industrial Pharmacy, College of Pharmacy and Drug Manufacturing, Misr University of Science and Technology (MUST), 6th of October, Giza, (12566) Egypt.; ^5^Department of Pharmacology and Toxicology, Faculty of Pharmacy, Fayoum University, Fayoum, Egypt.

**Keywords:** Coenzyme Q10, Hyperlipidemia, Selenium, Solid lipid nanoparticles, Simvastatin

## Abstract

**Purpose:**

The objective of this study was to develop a nanoencapsulated platform for coenzyme Q10 nanoparticles (coQNPs) or selenium nanoparticles (SeNPs) and explore their potential therapeutic benefits in treating hyperlipidemia and combating simvastatin (SV)-induced myopathy and adverse reactions in hyperlipidemic rats.

**Methods:**

The physical and chemical properties of the solid nanoparticles, coQNPs, and SeNPs were characterized, including zeta potential studies. Male Wistar albino rats were treated with various interventions for 112 days, including a nano-vehicle only, high-fat diet (HFD), HFD with SV alone, or with coQNPs or/and SeNPs for the last 30 days.

**Results:**

The coQNPs and SeNPs exhibited uniform spherical shapes with high encapsulation efficiency (EE% 91.20±2.14 and 94.89±1.54, respectively). The results demonstrated that coQNPs and SeNPs effectively reduced hyperlipidemia, insulin resistance, SV-induced myopathy, and hepatotoxicity. However, combining SV with coQNPs and SeNPs resulted in severe liver and muscle damage. Treatment with SV and SeNPs or SV and coQNPs alone showed significant improvements compared to SV treatment alone.

**Conclusion:**

These findings suggest that the CoQNPs or SeNPs platforms offer advanced relief for hyperlipidemia and insulin resistance while limiting adverse effects such as myopathy and hepatotoxicity.

## Introduction

 There is a rapid transition in dietary habits from healthy, homemade food to fast and junk food.^1^ This shift has increased metabolic diseases like hypocholesteremia, insulin resistance, and coronary heart diseases.^2^ Hypercholesterolemic patients are more likely to suffer from other cardiovascular diseases, which account for one-third of total deaths globally, with 17.8 million deaths in 2017.^3,4^ Simvastatin (SV) is the first-line treatment option for this condition. However, SV therapy may oppose several challenges due to its lower bioavailability, which is less than 5%. It is poorly absorbed from the gastrointestinal tract in some patients.^5^ Approximately 95% of an oral dose is not absorbed.^6^ In addition, statin treatment is associated with musculoskeletal damage and severe changes in liver function in up to 1-3% of patients.^7^ The long-term use of statins is associated with disruption of insulin signaling that may result in insulin resistance.^8,9^ No complete explanation can be used to answer the question related to the mechanism of statin-induced myopathy.^10^

 Earlier studies reported that statins-induced myopathy through inhibition of mitochondrial synthesis of Coenzyme Q10 (CoQ10), which ultimately interferes with respiratory chains and the capacity for energy production by mitochondria.^11^ CoQ10 is the most commonly fat-soluble vitamin, especially in the heart, liver, kidney, and brain.^12^ CoQ10 is a fat-soluble vitamin found mainly in the heart, liver, kidney, and brain.^13^ Selenium is a vital micronutrient with antioxidant activity, and its supplementation is prescribed for immune and neural-related diseases.^14^

 No conclusive data confirm that mitochondrial myopathy is because of the reduction of intramuscular levels of CoQ10.^15^ Supplementation of nutraceuticals and herbs may alleviate the side effects of different drugs in the conventional formulation.^16,17^ Nutraceuticals attract special attention from researchers, stakeholders, and consumers because of their potential effects on preventing or treating different pathological conditions.^15,18^ Nanotechnology may provide feasible solutions to improve the therapeutic efficacy of different drugs and nutrients, including nutraceuticals.

 This study aims to investigate the therapeutic potential of a nanoencapsulated platform containing CoQ10 or selenium for treating hyperlipidemia and evaluate their efficacy in mitigating SV-induced myopathy and adverse reactions in hyperlipidemic rats.

## Material and Methods

###  Material 

 Global Napi Pharmaceuticals, located in Giza, Egypt, provided SV, which was prepared by suspending it in distilled water at a concentration of 20 mg/kg.^19^ Arab Company for Pharmaceutical and Medicinal Plants in Cairo, Egypt, gifted CoQ10, while selenium powder was obtained from Egyptian European Pharmaceutical Industries in Alexandria, Egypt. Compritol 888 ATO (glyceryl behenate, a mixture of approximately 15% mono-, 50% di-, and 35% triglycerides of behenic acid) and Gelucire 40/14 (PEG glyceride) were generously provided by Gatteffose in France, and Poloxamer 407 was obtained from BASF in Florham Park, NJ. All other chemicals were purchased in high analytical grade from Sigma-Aldrich for Chemicals in St. Louis, MO, USA.

###  Experimental animals

 Wistar albino male rats weighing between 150-200 g were obtained from the laboratory animal farm of the National Research Center (Dokki, Giza, Egypt). After random assignment to experimental groups, the animals were housed in polycarbonate cages with a maximum capacity of 5 rats per cage and placed in a filter-top enclosure under well-controlled laboratory conditions, including a temperature of 25 ± 2 ºC, a 12/12 hours light-dark cycle, a diet consisting of rodent pellets, and free access to water ad libitum. The rats were allowed to acclimate to their new environment for a week before the start of the experimental work. All experimental procedures complied with the guidelines and code of ethics established by the Faculty of Pharmacy, Cairo University, Cairo, Egypt research ethics committee and adhered to the Laboratory Animals Welfare rules. The experimental design and procedure followed the requirements of the three Rs’ rules (Replace, Reduce, Refine). The animals were treated with care and gentleness to avoid squeezing, pressure, pain, malnutrition, abnormal cold or heat, injury, illness, and rough manipulations.

###  Development of drug-loaded solid lipid nanoparticles (SLNs)

 To develop drug-loaded SLNs, CoQ10 or selenium SLNs were prepared using the hot melt ultrasonication method, as previously reported.^9^ CoQ10 or selenium (0.5% w/v) was mixed with molten lipids (1% Compritol and 3.04% Gelucire) at 75 °C until the formation of a homogeneous mixture. A 3% w/v Poloxamer 407 solution was prepared in PBS (pH 7.4) at 75 °C. The poloxamer solution was gradually added to the molten lipid phase with homogenization (GLH 850, Omni Inc., USA) at 5000 to 15 000 rpm and 75 °C. The resulting suspension was sonicated (Model LC 60/H, Elma, Germany) for 3 minutes at 50% amplitude. The obtained nanosuspension was cooled until it reached room temperature and stored at 2-8 °C until further investigation.

###  Determination of particle size and zeta potential 

 The particle size and zeta potential were determined using a Malvern Zetasizer Nano ZS (Malvern Instruments, Malvern, UK), and all measurements were repeated at least three times to determine means and standard deviations.

###  Drug entrapment efficiency

 The entrapment efficiency (EE%) was measured using an indirect technique, where the unentrapped drug was measured in the supernatant after centrifugation (Model 3K 30, Sigma, Germany) at 18 000 rpm for 45 minutes at 4 °C. The free selenium ions in the supernatant were measured using a previously reported spectrophotometric method after complexation of selenium ions with 4,5-diamino-6-hydroxy-2-mercapto pyrimidine (DAHMP) at 458 nm using a spectrophotometer (Shimadzu UV 1650, Japan).^20^ Free CoQ10 was measured using HPLC (Agilent, USA) with a mobile phase of 1-propanol: methanol (60:40 v/v), a flow rate of 1.4 ml/min, and a wavelength of 275 nm.

###  Morphological study

 An electron microscope (Nova Nano SEM, FEI, USA) was used to study the morphological pattern of different particles, and the sample was prepared with gold sputtering for 20 seconds.

###  In vitro experiment 

 The dialysis bag diffusion technique was used for 24 hours using a dialysis membrane (Mw cutoff 12 kDa; Severa) to evaluate the in-vitro drug release profiles of CoQ10 or selenium.^9^

###  In vivo study

####  HFD preparation

 According to Onyeali et al,^21^ an HFD (20% w/w) was formulated by adding 200 g of unsaturated fats (margarine) to 800 g of the pellet. Unsaturated fats (margarine) contain vitamin A, vitamin D3, and antioxidants, according to the attached information on the pack. The HFD was dried in a dry place (22-25 °C) for 12 hours and stored in polyethylene or glass containers.

####  Experimental induction of hyperlipidemia by HFD

 Animals were assigned for daily ingestion of 20 g of HFD for 112 days to induce hyperlipidemia.^22^

###  Experimental study design

 Sixty male adult rats (8-10 rats/group) were randomly assigned into:

- Group I: Nano-vehicle control group was treated daily with oral preparation of the vehicle (20 mg/kg) for the last 30 days of the in vivo experiment.
- Group II (HFD): Rats were subjected to the HFD (20% of the daily required food) for 112 days.^21^
- Group III (HFD + SV): rats with hyperlipidemia and treated daily with oral formulation of SV (20 mg/kg) throughout the last 30 days.^23^
- Group IV (HFD + SV and coQNPs): rats with hyperlipidemia and treated daily with a combination of oral preparation of SV (20 mg/kg) and nano-CoQ10 (10 mg/kg) for the last 30 days of the in vivo experiment.^24^
- Group V (HFD + SV and SeNPs): hyperlipidemic rats treated with a combination of SV (20 mg/kg) and nano-selenium^25^ (0.1 mg/kg/d, PO) for the last 30 days
- Group VI: (HFD + SV + coQNPs and SeNPs): hyperlipidemic rats treated daily with a combination of SV (20 mg/kg), nano-CoQ10 (10 mg/kg) and nano-selenium (0.1 mg/kg) for last 30 days of the *in vivo* experiment.

Every week, the changes in animal body weight were calculated. The *in vivo* study was terminated by fasting animals overnight after delivering the last dose of the treatment regimen. Animals were treated with ketamine (12.5 mg/kg) and xylazine (1.5 mg/kg) for anesthesia. The blood samples were collected using non-heparinized microhematocrit capillary tubes from the retro-orbital vein.^26^ Blood samples were collected for serum separation by cooling centrifugation at 3000 rpm for 15 minutes and stored at -20 °C until analysis. The sera were used for the estimation of serum level of total cholesterol (TC), triglycerides (TG), high-density lipoproteins cholesterol (HDL-c), glucose, creatine kinase (CK), creatinine, urea, alanine aminotransferase (ALT), aspartate aminotransferase (AST), albumin, and alkaline phosphatase (ALP) (Biomed Diagnostics, Egypt), insulin (DRG International, Inc., USA), troponin and myoglobin (Cusabio biotech co., Wuhan, China) according to the instructions of the analytical kits. Immediately after collecting the blood samples, an overdose of anesthesia euthanized animals, and the liver and quadriceps muscles were detached and distributed into two portions. The first portion of liver tissue was prepared for homogenization by tissue homogenizer (Heidolph, DIAX 900, Germany) to obtain (20% w/v). The supernatant was separated from the homogenate by centrifugation for 15 minutes (4000 rpm at 4 °C). These supernatants were used for the estimation of malondialdehyde (MDA) level, glutathione (GSH), and superoxide dismutase (SOD) activities (Biodiagnostics, Cairo, Egypt) according to the instruction of the kits. The other part of the liver and quadriceps muscle was mounted in 10% formalin in saline for histological and immunohistochemical analysis.

###  Statistical analysis

 The results of the current study were articulated as means ± standard error of the mean (SE). GraphPad software was used for statistical analysis (software 2003, version 3.06 Inc., San Diego, USA). One-way analysis of variance (ANOVA) followed by Dunnett’s multiple comparisons test has been used to determine whether a statistical difference has existed among different groups or not.^27^ One-way ANOVA was used to analyze rats’ monthly total body weight (TBW), and then Tukey’s multiple comparisons test was performed. All statements of significance were based on the probability of *P* ˂ 0.05.

## Results and Discussion

###  Particle size and polydispersity indexes and zeta potential

 Nanomedicine is a vital approach to improve drug use and enhance the safety of most existing drugs.^28^ However, there are several hurdles in translating nanomedicine from bench to bedside.^29^ SLNs were prepared by the hot homogenization/sonication method by emulsifying solid lipids with a poloxamer solution. Table 1 shows the successful preparation of blank SLNs with particle size 250.2 nm and zeta potential -17.3 mV. Encapsulation of CoQ10 slightly decreased particle size to reach 213.9 nm and zeta-potential of -13.49 mV, while encapsulation of selenium increased particle size to reach 296.72 nm and zeta potential of -6.12 mV.

**Table 1:** Characterization of unloaded and loaded solid lipid nanoparticles

**Formulation**	**Particle Size (nm)**	**PDI**	**Zeta-potential (mV)**	**EE (%)**
Blank SLNs	250.2 ± 5.2	0.52 ± 0.10	-17.30 ± 1.95	-
Coenzyme Q10-SLNs	213.9 ± 6.3	0.35 ± 0.15	-13.49 ± 3.85	91.20 ± 2.14
Selenium SLNs	296.72 ± 6.3	0.49 ± 0.12	-6.12 ± 2.85	94.89 ± 1.54

###  Drug entrapment efficiency

 The EE of coenzyme Q 10 was 91.2%, which was measured indirectly. On the other hand, selenium encapsulation increased particle size to 296.72 nm, while zeta potential was decreased to -6.12 mV due to the adsorption of selenium ions on the SLN surface. The spectrophotometric method measured the EE percentage, where 94.89% was entrapped inside SLNs (Table 1).

###  Morphological study

**Figure 1:**
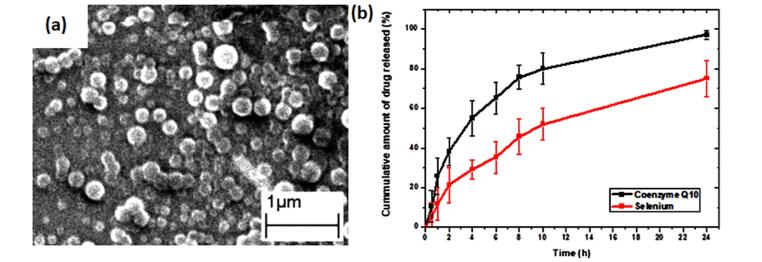
Scanning electron microscope image of the blank solid lipid nanoparticles

 SLNs were visualized using SEM to investigate particle morphology (Figure 1a). SEM micrograph demonstrated spherical shape particles. The particle size from SEM images showed a relevant range aligned with the reported particle size using Zetasizer. The combination of homogenization and sonication reduced the particles to reach nano size. Blank nanoparticles were prepared based our previous report, where1% w/v Compritol, 3% w/v Gelucire® 44/14, and 3% w/v poloxamer 407 were used as optimum parameters^9^

###  In vitro drug release test

 The *in vitro* release study of both drugs was conducted in PBS. CoQ10 showed a rapid release profile, where ~85% was released in 12 hours. On the other hand, selenium released showed a slower profile, where ~50% was released in 12 hours and ~75% was released in 24h. It could be attributed to interaction with the electrostatic charge obtained from selenium ions and negatively charged lipid nanoparticles (Figure 1b).

###  In vivo study

####  Effect of SV, nano-CoQ10, and/or nano-selenium on TBW in hyperlipidemic rats 

**Figure 2:**
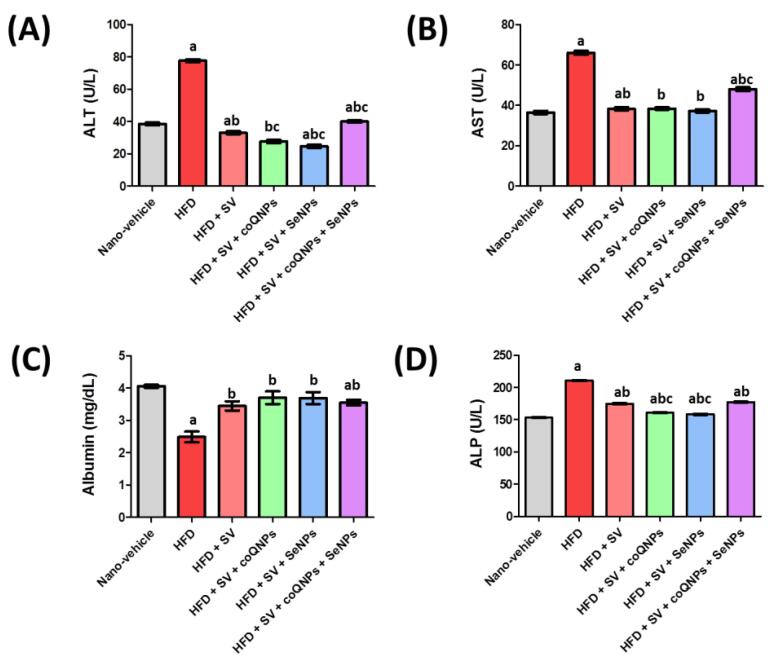
Effect of simvastatin (20 mg/kg) and its combination with nano-CoQ10 (10 mg/kg) and/or nano-selenium (0.1 mg/kg) on (A) ALT level, (B) AST level, (C) Albumin level, (D) ALP.Data is described as means ± SEM (n = 8 rats). ^a^ Significantly different from nano-vehicle value at *P* < 0.05; ^b^ Significantly different from HFD value at *P* < 0.05; ^c^ Significantly different from simvastatin value at *P* < 0.05

 Animals treated with HFD exhibited a prominent increment of ΔTBW by 29.9% compared to animals treated with a nano-vehicle group. Meanwhile, treatment with SV, co-administration of SV and coQNPs, concurrent administration of SV and SeNPs, and combined therapy of SV and coQNPs and SeNPs resulted in a significant decrease in ΔTBW by 48.6%, 51.0%, 61.8% and 60.9%, respectively, in comparison to HFD treated rats (Figure 2). Fortunately, all combination groups reduced ΔTBW by 4.6%, 25.7% and 24.0%, respectively in comparison to SV treated animals (Table 2).

**Table 2:** Effect of simvastatin (20 mg/kg) and its combination with nano-CoQ10 (10 mg/kg) and/or nano-selenium (0.1mg/kg) on body weight, lipid profiles and AIX in hyperlipidemic rats

**Groups**	**Parameters **					
	**Total BW difference (ΔTBW)**	**TC (mg/dL)**	**Triglycerides (mg/dL)**	**HDL-c (mg/dL)**	**LDL-c (mg/dL)**	**AIX**
Nano-vehicle	162.4 ± 4.7	163.3 ± 0.9	86.5 ± 0.86	50.48 ± 0.95	95.5 ± 1.16	2.24 ± 0.06
HFD	210.9 ± 0.28 ^a^	224.3 ± 0.96^a^	140.1 ± 0.79^a^	18.89 ± 0.75^a^	177.4 ± 1.57^a^	11.02 ± 0.54^a^
HFD + SV	108.4 ± 0.29 ^ab^	170.4 ± 0.96^ab^	108.1 ± 0.99^ab^	46.21 ± 0.98^ab^	102.5 ± 1.09^ab^	2.69 ± 0.07^b^
HFD + SV + coQNPs	103.4 ± 1.2 ^abc^	155.3 ± 0.9^abc^	90.36 ± 0.98^abc^	48.01 ± 0.64^b^	89.17 ± 1.08^abc^	2.24 ± 0.04^b^
HFD + SV + SeNPs	80.5 ± 0.78 ^abc^	154.9 ± 0.74^abc^	89.86 ± 0.97^bc^	50.64 ± 0.96^bc^	86.27 ± 1.06^abc^	2.07 ± 0.05^b^
HFD + SV + coQNPs + SeNPs	82.4 ± 0.98 ^abc^	153.5 ± 0.95^abc^	92.16 ± 0.86^abc^	51.29 ± 0.98^bc^	83.24 ± 1.66^abc^	2.0 ± 0.07^bc^

Data is described as means ± SEM (n = 8 rats); ^a^ Significantly different from nano-vehicle value at *P* < 0.05; ^b^ Significantly different from HFD value at *P* < 0.05; ^c^ Significantly different from simvastatin value at *P* < 0.05.

####  Effect of SV, nano-CoQ10, and/or nano-selenium on lipid profiles and atherogenic index (AIX) in hyperlipidemic rats

 Alteration in lipid profile markers was depicted after daily consumption of HFD for 112 days. Thus, a remarkable elevation in serum level of TC was noticed in hyperlipidemic rats by 37.4% compared to a nano-vehicle control group. While co-administration of either SV alone or in combination with coQNPs or/and SeNPs resulted in enhancement in the anti-hypercholesteremic efficacy. Moreover, co-administration of SV and coQNPs, co-administration of SV and SeNPs, and combination therapy of SV and coQNPs and SeNPs provoked a more pronounced reduction in serum TC levels 8.9%, 9.1%, and 9.9%, respectively, as compared to SV treated group. Animals treated with HFD showed a marked rise in serum TG level by 62.0% compared to the nano-vehicle group. The combined therapy of SV and SeNPs reached the average value of TG level and showed a significant reduction of TG by 16.9% compared to the SV-treated group (Table 2). Treatment with SV plus coQNPs and SeNPs showed a marked increase in serum HDL-c level by 171.5%, respectively, relative to the HFD control group. HFD distinctly augmented serum low-density lipoprotein cholesterol (LDL-c) level by 85.8%. At the same time, the combined therapy of SV, coQNPs, and SeNPs showed a significant decrease in serum LDL-c levels by 42.2%, 49.7%, 51.4%, and 53.1%, respectively, compared to HFD-treated rats. Hyperlipidemic rats showed a prominent increase in AIX ratio by 392.0%. In comparison, such increment in AIX was markedly decreased by treatment with SV alone or in combination with coQNPs or/and SeNPs by 75.6%, 79.9%, 81.2%, and 81.9%, respectively. Furthermore, all combination treatments successfully restrained the typical ratio of AIX (Table 2).

 In the present work, HFD was associated with severe alterations in lipid profile biomarkers manifested by marked elevation in the AIX, TC, TG, and LDL-c in addition to a deficiency of HDL-c. The outcomes of the current study were in the same context as earlier experiments.^30,31^ The results align with liver histopathological findings, which noticed a diffusion of fatty droplets that accompanied all hepatocytes^32^ The present investigation showed that HFD induced a marked elevation of liver function tests; ALT, AST, and ALP were associated with a marked reduction in albumin levels. Thus indicating hepatic steatosis and liver injury, and this finding was in line with many previous studies.^33,34^ The current study showed that atrophied muscles were filled with fatty droplets and Zenker’s necrosis in other bundles. The histological changes were in the same line as earlier studies.^32^ Animals treated with HFD showed muscular dysfunction indicated by a significant elevation of serum CK, troponin, and myoglobin levels. It has been well reported that consumption of an HFD was associated with impairment of the alternative splicing process of pre-mRNA of troponin-T with stimulation of protein expression of myoglobin and activation of CK enzymes of skeletal muscles.^35^ The present work showed that SV (20 mg/kg) treated rats exhibited a distinct drop in lipid profile, including TC, TG, and LDL-c. These results were consistent with the previous work.^36^ The current results showed that the combination of SV and coQNPs exhibited a significant decrease in serum LDL-c, TC, and TG with a significant increment in HDL-c and a normalized AIX. These findings were in line with previous studies^37,38^

 Moreover, clinical research reported that endothelial function was improved in dyslipidemic patients who used CoQ10 regularly due to anti-atherogenic activities, especially in conduit arteries.^39^ Earlier studies reported that CoQ10 can enhance mitochondrial fatty acid oxidation and vascular protection by excessive degradation of T.G-rich lipoprotein. In the present work, the combination therapy of SV and SeNPs exhibited a pronounced anti-hyperlipidemic activity relative to SV alone associated with a marked reduction in liver function biomarkers, which is in line with previous work that speculated on the improvement of SeNPs on hepatic disturbances, histopathology, and oxidative stress, which evidenced by our histopathological and immunohistochemical findings.^40^

####  Effect of SV, nano-CoQ10, and/or nano-selenium on hepatic function tests, albumin, and ALP in hyperlipidemic rats

 Hyperlipidemic rats significantly elevated serum ALT activity by 155.5% compared to the nano-vehicle-treated group. Moreover, SV alone or combined with coQNPs/ or SeNPs and combined therapy of SV and coQNPs and SeNPs leads to a significant decline in serum ALT activity. HFD markedly blunted serum AST activity by 84.6% compared to a nano-vehicle control group. The combined treatment of SV and SeNPs showed a significant decrease in AST of 44.9%, respectively, compared to the HFD-treated group. Hyperlipidemic rats showed a marked reduction in serum albumin, and co-administration of SV and coQNPs/ or SeNPs normalized the serum albumin level compared to the nano-vehicle-treated group. HFD exhibited a significant elevation in serum ALP.

 Meanwhile, treatment with SV alone or combined with coQNPs or/and SeNPs revealed a significant decrease in serum ALP activity by 17.0%, 23.6%, 24.9%, and 15.8%, respectively, compared to a hyperlipidemic control group. Furthermore, it was observed that co-administration of SV and coQNPs and concurrent administration of SV and SeNPs had a better effect on serum ALP activity by 8.0% and 9.6%, respectively, as compared to the SV-treated group. The results were graphically illustrated in Figure 2. Hepatic injury was manifested by a significant increment in serum levels of ALT, AST, ALP, and reduction of albumin due to SV treatment. Previous studies exhibited that SV treatment was associated with hepatitis, cholestatic jaundice, cirrhosis, hepatic failure, and hepatic necrosis, indicating the destruction of hepatic tissues.^41^ It agreed with our finding in histopathological examination that showed progressive parenchymal liquefied degeneration in addition to limited infiltration of inflammatory cells around the portal vein. Furthermore, SV and coQNPs reduced ALP activity with normalized ALT, AST, and albumin with a distinct decrease of CK activity, MYO, and troponin relative to S.V. CoQ10 reduces statin side effects and has an anti-obesity effect.^42^

####  Effect of SV, nano-CoQ10, and/or nano-selenium on insulin, blood glucose, and kidney function test in hyperlipidemic rats

 Serum glucose level was augmented in hyperlipidemic rats by 104.3% compared to nano-vehicle-treated rats. Treatment with SV alone or combined with coQNPs or/and SeNPs showed a significant decrease in glucose by 44.8%, 49.4%, 42.3%, and 44.4%, respectively, compared to the HFD-treated group. However, Hyperlipidemic rats showed a significant elevation in serum insulin level by 56.3% relative to a nano-vehicle control group. Such an increase was markedly hampered by treatment with SV alone or in combination with coQNPs or/and SeNPs, leading to a pronounced reduction in insulin levels. Elevation in serum creatinine level was revealed after induction of hyperlipidemia. While treatment with either SV alone or with coQNPs or/and SeNPs showed a significant reduction in serum creatinine. SV and coQNPs or SeNPs succeeded in normalizing serum creatinine levels. Hyperlipidemic rats revealed a marked spike in serum urea level; meanwhile, treatment with SV, co-administration of SV, and coQNPs or/and SeNPs showed a significant decrease in urea level in hyperlipidemic rats. Combined treatment of SV with coQNPs or SeNPs was observed to have restored the standard serum urea level compared to the nano-vehicle group. The HFD group significantly blunted the BUN ratio, but this increase in BUN was markedly hampered by treatment with SV individually or in the presence of coQNPs or/and SeNPs. It was observed that co-administration of SV and coQNPs or SNPs showed a significant reduction in BUN ratio by 50.6% and 52.6%, respectively (Table 3). Biochemical examination of insulin and blood glucose levels revealed that animals treated with HFD exhibited marked elevation of both biomarkers. These findings may be attributed to the masking effect of HFD on insulin receptors and the promotion of insulin resistance. HFD potentiates the key cytokines such as IκB kinase, cJun-N-terminal-kinase, and protein kinase C that promote insulin resistance and interference with insulin signaling.^43^ The data of the present work showed that HFD induced severe alterations of kidney function biomarkers via severe elevation in all indicators, including creatinine, urea, and BUN ratio. These results were affirmed by outcomes that showed HFD activates PI3K/Akt and mitogen-activated protein kinase, leading to renal impairment and glomerular hypertrophy.^44^ In the present work, blood glucose levels and insulin were significantly decreased by SV treatment in animals pretreated with HFD. These results agreed with previous work,^45^ which reported the inhibitory role of statins on dipeptidyl peptidase IV (DPP-IV). DPP-IV is a serine protease enzyme that catalyzes the decomposition of glucagon-like peptide 1 (GLP-1) and glucose-dependent insulin-tropic polypeptide (GIP). GLP-1 and GIP are vital in maintaining normal blood glucose levels by preserving 70% of insulin secretion after meals. All kidney function biomarkers such as creatinine, urea, and BUN were increased in SV- the treated group, compared to corresponding control groups. It has been well-reported that renal failure is most likely associated with rhabdomyolysis.^46^ Consistent results highlight the relationship between statin therapy and the reduction of serum levels of CoQ10.^47^ There are mounting shreds of evidence that link between deficiency of CoQ10 on one side and skeletal muscle dysfunction and insulin resistance on another side due to the essential role of skeletal muscles in glucose uptake and insulin regulation.^48^

**Table 3:** Effect of simvastatin (20mg/kg) and its combination with nano-CoQ10 (10mg/kg) and/or nano-selenium (0.1mg/kg) on insulin, blood glucose levels and kidney function test in hyperlipidemic rats

**Group **	**Parameters**				
	**Insulin (µIU/mL)**	**Glucose (mmol/L)**	**Creatinine (mg/dL)**	**Urea (mg/dL)**	**BUN (mg/dL)**
Nano-vehicle	14.4 ± 0.55	8.41 ± 0.6	0.99 ± 0.021	34.59 ± 0.98	16.2 ± 0.46
HFD	22.5 ± 0.95^a^	17.18 ± 0.5^a^	2.01 ± 0.038^a^	116.0 ± 0.91^a^	54.2 ± 0.43^a^
HFD + SV	15.71 ± 0.51^b^	9.48 ± 0.5^b^	1.42 ± 0.084^ab^	77.11 ± 1.04^ab^	36.03 ± 0.49^ab^
HFD + SV & coQNPs	15.46 ± 0.68^b^	8.69 ± 0.36^b^	1.07 ± 0.034^bc^	38.1 ± 0.095^bc^	17.8 ± 0.44^abc^
HFD + SV & SeNPs	14.55 ± 0.53^b^	9.91 ± 0.47^b^	1.02 ± 0.035^bc^	36.56 ± 0.84^bc^	17.09 ± 0.4^abc^
HFD + SV & coQNPs & SeNPs	14.31 ± 0.92^b^	9.55 ± 0.41^b^	1.5 ± 0.067^ab^	79.59 ± 1.01^ab^	37.19 ± 0.47^ab^

Data is described as means ± SEM (n = 8 rats); ^a^ Significantly different from nano-vehicle value at *P* < 0.05; ^b^ Significantly different from HFD value at *P* < 0.05; ^c^ Significantly different from simvastatin value at *P* < 0.05.

 This data would explain our results that serum glucose and insulin levels were normalized by administration of the combined therapy of SV and coQNPs.

####  Effect of SV, nano-CoQ10, and/or nano-selenium on oxidative stress biomarkers in hyperlipidemic rats

 Increased inflammatory response was depicted after daily consumption of HFD, evidenced by a remarkable elevation in liver MDA content. Treatment with SV in combination with coQNPs or/and SeNPs exhibited a marked reduction in the MDA level of hepatic tissues. Treatment withSV and coQNPs or/and SeNPs revealed a distinct decrease in liver MDA content by 60.4% and 14.5%, respectively, compared to the SV-treated group. Furthermore, concurrent administration of SV and SeNPsshowed restoration of the normal liver MDA content and a significant reduction of 63.5% compared to the treated group. Hyperlipidemic rats showed a marked depletion in liver GSH content. However, it was replenished by treatment with SV alone or in combination with coQNPs or/and SeNPs, resulting in a significant elevation in liver GSH. Treatment with SV and coQNPs or SeNPs restored liver GSH content to normal. A marked reduction in liver SOD activity was noticed in hyperlipidemic rats.

 Meanwhile, treatment with SV in combination with coQNPs or/and SeNPs showed significant elevation in liver SOD activity (Table 4). HFD consumption precipitates oxidative stress that indicates to series of inflammatory reactions associated with the promotion of cytokines such as nuclear factor κB, activation of genetic expression of inducible nitric oxide synthase and cyclooxygenase-2.^49^ SV treatment showed a pronounced improvement in antioxidative stress biomarkers. These outcomes agreed with previous studies that revealed statin treatment associated with attenuation of lipid peroxidation.^50^ Meanwhile, clinical research showed a positive impact of SV treatment in hypercholesterolemic and diabetic patients, but without benefits on healthy volunteers.^51^ This antioxidant activity of coQNPs was compatible with our results. Thus, it was found that a combination regimen of SV and coQNPs reduced MDA significantly with a normalized level of GSH and SOD.^52^ Another vascular protecting mechanism was shown by limiting the inactivation of endothelial NO in response to superoxide radicals.^53^ Therefore, the inactivation of endothelium-derived relaxing factor and/or fibrosis of arteriolar smooth muscle will be reduced.^54^ The most apparent effect of the combination group of SV and SeNPs is the restoration of the oxidative stress biomarkers. The previous study stated that selenium could scavenge free radicals and terminate oxidative stress through selenoenzymes such as glutathione peroxidase.^55^

**Table 4:** Effect of simvastatin (20 mg/kg) and its combination with nano-CoQ10 (10 mg/kg) and/or nano-selenium (0.1 mg/kg) on oxidative stress biomarkers in hyperlipidemic rats

**Group**	**Parameter**		
	**MDA (nmol/mg)**	**GSH (mmol/mg)**	**SOD (u/g)**
Nano-vehicle	19.13 ± 0.42	49.79 ± 0.9	2.095 ± 0.14
HFD	113.1 ± 0.89^a^	19.49 ± 0.2^a^	0.32 ± 0.06^a^
HFD + SV	58.1 ± 0.96^ab^	38.56 ± 1.35^ab^	1.82 ± 0.14^b^
HFD + SV & coQNPs	23.0 ± 1.07^abc^	49.11 ± 1.29^bc^	2.07 ± 0.18^b^
HFD + SV & SeNPs	21.2 ± 0.87^bc^	49.17 ± 0.81^bc^	2.03 ± 0.18^b^
HFD + SV & coQNPs & SeNPs	49.7 ± 1.0^abc^	39.0 ± 0.98^ab^	2.11 ± 0.12^b^

Data is described as means ± SEM (n = 8 rats); ^a^ Significantly different from nano-vehicle value at *P* < 0.05; ^b^ Significantly different from HFD value at *P* < 0.05; ^c^ Significantly different from simvastatin value at *P* < 0.05.

####  Effect of SV, nano-CoQ10, and/or nano-selenium on skeletal muscle function biomarkers in hyperlipidemic rats

 The effect of SV, nano-CoQ10, and/or nano-selenium on skeletal muscle function biomarkers in hyperlipidemic rats was markedly augmented. The serum CK activity was reversed by co-administration of SV and coQNPs or SeNPs, causing a considerable reduction in serum CK activity by 11.3% and 16.1%, respectively. Paradoxically, treatment with SV besides coQNPs and SeNPs showed a significant elevation in serum CK activity of 6.1% compared to the SV-treated group. Hyperlipidemic rats provoked a pronounced spike in serum myoglobin (MYO) level by 523.5% compared to the nano-vehicle group. While treatment with SV with coQNPs or/and SeNPs showed a significant decrease in serum MYO level. Unfortunately, combined treatment of SV with coQNPs and SeNPs showed a marked rise in serum MYO level by 29.6%. Serum troponin (Tn-T) level was significantly elevated in hyperlipidemic rats.

 On the other hand, Tn-T was notably lowered by the treatment with SV alone or in combination with coQNPs or/and SeNPs. Furthermore, concurrent administration of SV and SeNPs ultimately restrained the average serum Tn-T level with a significant reduction of 45.2% compared to the SV-treated group (Table 5). Laboratory findings of the present study revealed that animals fed HFD and treated with SV hyperlipidemic rats suffered from myopathy that was manifested by a significant increment of serum CK, myoglobin, and troponin compared to standard groups. There needs to be a complete explanation of the mechanism of statin-induced rhabdomyolysis. However, previous studies speculated on the role of monocarboxylate transported in uptaking and accumulation of stains in skeletal muscles,^56^ besides deterioration of cell membrane fluidity and stability because of shortage of membrane cholesterol^57^ that is associated with dysfunction of chloride channel conductivity versus cytoplasmic overload due to malfunction of mitochondria.^58^ The research outcome of an earlier study revealed that patients treated with statins for a long time suffer from muscle aches due to a deficiency of mevalonate and isoprenoids.^59,60^ Indeed, mevalonate deficiency will open the door in front of unwanted reactions such as lack of potent antioxidant, cell membrane destabilization due to CoQ10/ubiquinone insufficiency,^61^ lack of enzymatic activity of isoprenylation of selenocysteine-tRNA because of shortage of selenoproteins resources,^62^ inactivation of the prenylation/geranylgeranylation of proteins through reducing in farnesyl and geranylgeraniol pyrophosphates^61,63,64^ and agitation of N-linked glycosylation within proteins.^65^ Consequently, fat cell deposition within quadriceps muscles and atrophied cells of histopathological studies further explains this phenomenon. A study by Abdelbaset et al^66^ repurposed CoQ10 in lowering cholesterol levels without elevation of CK compared to statin-induced myopathy. These results were confirmed by the histological and immunohistochemical studies in the present work. In the current study, the combined therapy of SV and SeNPs restored the standard glucose and insulin values. Earlier studies advocate selenium use in diabetes due to activation of Akt and other kinases that control insulin signaling and carbohydrate metabolism. Several explanations exist for the hypoglycemic action of selenium, including prevention of glucose transport from the GIT tract and enhancement of urinary excretion.^67^ Furthermore, selenium improves insulin sensitivity and glucose transport in experimental animal models of diabetes.^68^

**Table 5:** Effect of simvastatin (20 mg/kg) and its combination with nano-CoQ10 (10 mg/kg) and/or nano-selenium (0.1 mg/kg) on skeletal muscle function biomarkers in hyperlipidemic rats

**Group **	**Parameters**		
	**CK (Creatine kinase) (U/L)**	**Myoglobin (ng/ml)**	**Troponin-T (pg/ml)**
Nano-vehicle	139.0 ± 0.8	7.025 ± 0.47	7.7 ± 0.61
HFD	226.1 ± 0.95^a^	43.8 ± 1.5^a^	49.86 ± 2.1^a^
HFD + SV	189.9 ± 0.97^ab^	15.9 ± 0.57^ab^	18.66 ± 0.91^ab^
HFD + SV & coQNPs	168.38 ± 0.98^abc^	13.8 ± 0.49^ab^	15.19 ± 0.47^abc^
HFD + SV & SeNPs	159.3 ± 1.03^abc^	8.8 ± 0.36^bc^	10.23 ± 0.3^bc^
HFD + SV & coQNPs & SeNPs	201.4 ± 1.08^abc^	20.6 ± 0.55^abc^	21.86 ± 0.62^abc^

Data is described as means ± SEM (n = 8 rats); ^a^ Significantly different from nano-vehicle value at *P* < 0.05; ^b^ Significantly different from HFD value at *P* < 0.05; ^c^ Significantly different from simvastatin value at *P* < 0.05.

 All the previously mentioned studies showed that SeNPs have lower side effects, manifested by normalized muscle function biomarkers and kidney function. The muscular histopathological findings showed a regular structure with lesser caspase-3 immunoreactivity than SV. It was noted that SeNPs possess equal efficacy of selenium with much lower toxicity.^69^

####  Histopathological and immunohistochemical examinations

**Figure 3:**
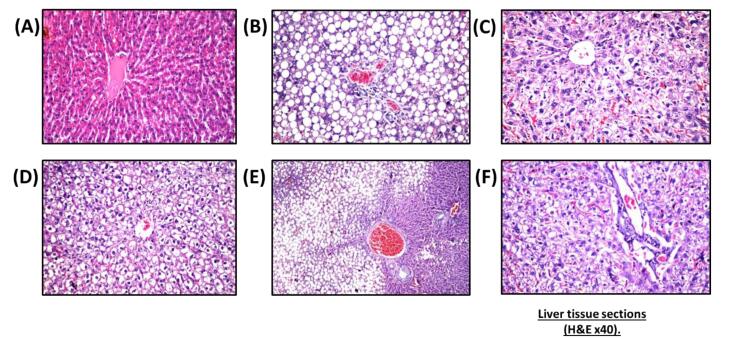
Histopathological patterns of liver tissues from different experimental groups. (A) nano-vehicle control group showing slight congestion round central vein and blood sinusoids, (B) HFD treated rat showing congestion in portal vein with migration of small number of inflammatory cells, infiltration in portal area and fatty change in the hepatocyte all in the hepatic parenchyma, (C) SV treated group showing diffuse and proliferation of Kupffer in between the degenerated hepatocytes with decongested hepatic sinusoids, (D) treatment with SV and coQNPs showing parenchymal vacuolar degeneration diffusion, (E) combination group of SV and SeNPs showing congestion in portal vein with focal fatty change in hepatocytes, (F) combination group of SV and coQNPs and SeNPs showing vacuolar degeneration in the hepatocyte and slight infiltration of limited number of inflammatory cell around portal vein (H&E × 40)

 Histological studies revealed that nano-vehicle control rats exhibited typical architecture of the central portal veins and blood sinusoids with a slight congestion (Figure 3A). HFD showed deterioration of hepatic tissues characterized by deposition of fatty droplets in the parenchyma associated with few inflammatory cells’ infiltration in the portal area as well as congestion in the portal veins and sinusoids (Figure 3B). A cross-section of hepatic tissue of SV-treated rats revealed vacuolar degeneration that was detected diffusely all over the hepatocytes in the parenchyma with few inflammatory cells’ infiltration in the portal area (Figure 3C). The liver tissue section of the combination group of SV and coQNPs showed vacuolar degeneration was noticed diffusely all over the hepatocytes in the parenchyma (Figure 3D). SV and SeNPs treatment exhibited a fatty change in the hepatocytes in a focal manner associated with portal vein congestion (Figure 3E). SV treatment in combination with coQNPs and SeNPs resulted in vacuolar degeneration in the hepatocytes associated with a few inflammatory cell infiltration in the portal area (Figure 3F).

**Figure 4:**
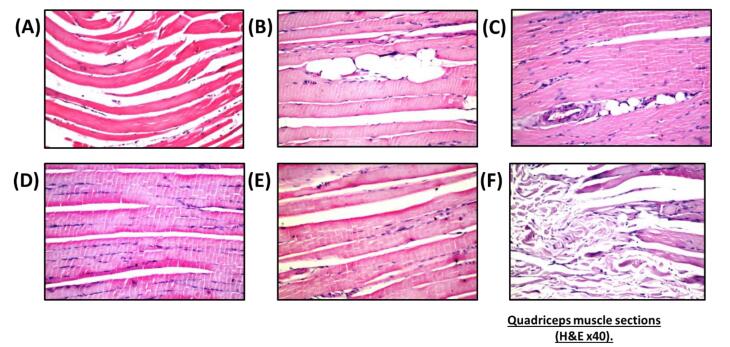
Effect of different treatments on quadriceps muscle sections.(A) nano-vehicle control group showing typical architecture of quadriceps muscle, (B) HFD treated group showing deposition of fatty droplets within atrophied muscle bundles with marked Zenker’s necrosis, (C) SV treated group showing focal deposition of the fat cells in between the muscle bundles with atrophied cells, (D) SV & coQNPs treatment showing no histopathological alterations, (E) combined therapy of SV & SeNPs showing no histopathological alterations, (F) concurrent administration of SV & coQNPs & SeNPs showing muscular atrophy with deposition of fatty droplets (H&E × 40)

 Nano-vehicle control rats showed no histopathological alterations in their quadriceps muscle (Figure 4A). HFD showed deposition in fat droplets between the atrophied muscles (Figure 4B) that was characterized by focal Zenker’s necrosis in a few other bundles. Treatment of SV showed marked deposited fat cells in the area between the muscle and atrophied cells (Figure 4C). Similarly, the quadriceps muscle section of rats treated with a combination group of SV and coQNPs showed typical muscle structure (Figure 4D). The SV and SeNPs combination group showed no histopathological alterations (Figure 4E). Furthermore, Combination therapy of SV, coQNPs, and SeNPs showed muscular atrophy, which was detected in a focal manner associated with focal deposition of the fat droplets between the bundles (Figure 4F). Histological changes in sections of hepatic tissues and quadriceps muscles were quantitatively illustrated in Table 6.

**Table 6:** The effect of different treatments on histological patterns of hepatic tissues and quadriceps muscles

**Organs**	**Histological changes **	**Groups**					
		**Nano-vehicle**	**HFD**	**HFD+SV**	**HFD+SV+coQNPs**	**HFD+SV+SeNPs**	**HFD+SV+coQNPs+SeNPs**
Hepatic tissues	Fatty droplets in hepatic tissues	-	+ + +	-	+ +	-	-
	liquefaction degeneration	-	-	+ + +	-	+ + +	+ +
	Migration of inflammatory cells	-	+	+	-	-	-
	Congestion	-	+	-	+ +	-	+
	Kupffer cells proliferation	-	-	+	-	-	+
Quadriceps muscle	Muscular atrophy	-	+ +	+	-	-	+ +
	Zenker’s necrosis	-	+ +	-	-	-	-
	Fat deposition (muscle bundles)	-	+ +	+ +	-	-	+ +

+ + + Severe, + + moderate, + mild, - nil.

**Figure 5:**
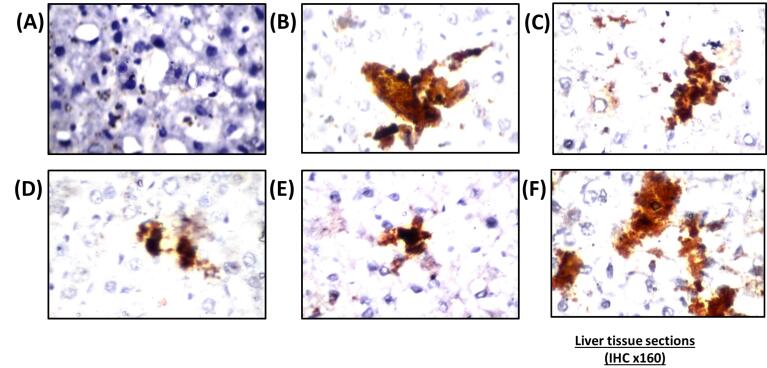
Immunoreaction of histochemical stain of caspase-3 in hepatic tissues. (A) Cross section of hepatic tissue of a nano-vehicle treated rat receiving showing negative reaction all over the tissue, (B) HFD treated rat showing an intense positive result (positive results represented dark brown discoloration) in many hepatocytes donating an increase in caspase immunoreactivity, (C) Photomicrograph of a hepatic section of SV treated rat showing an intense positive result, (D) Photomicrograph of a section of hepatic tissue of combination group of SV & coQNPs then stained immunohistochemically for caspase-3 showing reduction in number and intensity of positive cells, (E) Photomicrograph of a section of hepatic tissue of combination group of SV & SeNPs then stained immunohistochemically for caspase-3 showing reduction in the intensity of positive cells, (F) Cross section of hepatic tissue of concurrent administration of SV & coQNPs & SeNPs then stained immunohistochemically for caspase-3 showing an increase in number and intensity of positive results of hepatocytes. (H&E × 160)

 Photomicrograph of a section of hepatic tissue of rats stained with caspase-3, animals treated with nano-vehicle showed no interaction between hepatic tissues after staining with caspase 3 (Figure 5A). Photomicrograph of a hepatic section of rat treated with HFD stained immunohistochemically for caspase-3 showed an intense positive result (discoloration with dark brown) in many hepatocytes due to immunoreaction after staining with caspase 3 (Figure 5B). SV-treated rats showed an intense positive result indicated by dark brown discoloration (Figure 5C). The combined treatment with SV and coQNPs showed a significant reduction in positive cells stained with caspase-3 (Figure 5D). Photomicrograph of a section of hepatic tissue of combined therapy of SV and SeNPs shows a reduction in the intensity of positive cells compared to SV-treated rats (Figure 5E). Photomicrograph of a section of hepatic tissue of concurrent administration of SV and coQNPs and SeNPs then stained immunohistochemically for caspase-3 showing an increase in number and intensity of positive results of hepatocytes as compared to SV treated rat (Figure 5F).

**Figure 6:**
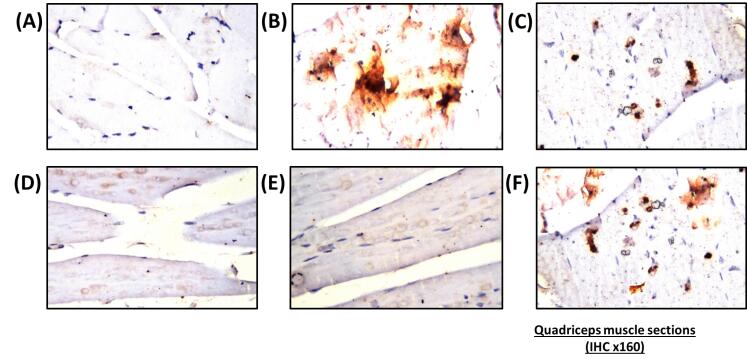
Histopathological examination of caspase-3 localization in rats’ quadriceps. (A) Cross section of quadriceps muscle of a nano-vehicle treated rat showing no negative result all over the tissue, (B) Quadriceps muscle of a rat fed with HFD that stained with caspase-3 showing an intense positive result due to increase in caspase immunoreactivity (positive results represented by dark brown color), (C) Simvastatin treated rat showing an intense positive result after staining with caspase-3, (D) Immunohistochemical for caspase-3 of quadriceps muscle removed from animals treated with simvastatin and nano-CoQ10 showing a decline in number and intensity of positive cells, (E) Combined treatment of simvastatin and nano-selenium showing a reduction in number and intensity of positive cells, (F) Simvastatin nano-selenium and nano-CoQ10 treated rat showing an increase in number and intensity of positive

 Animals treated with nano-vehicle only showed no reaction with caspase 3, indicated by negative results of quadriceps muscles (Figure 6A). The cross-section of the quadriceps muscle of animals treated with HFD exhibited a marked positive reaction with immunohistochemical stain caspase 3, indicated by dark brown discoloration in tremendous numbers of muscle filaments (Figure 6B). Immunohistochemical staining with caspase 3 showed distinct dark brown discoloration of the quadriceps muscle of animals treated with SV (Figure 6C). The cross-section of immunohistochemical strained quadriceps muscles with caspase 3 removed from animals treated with a combination of SV and coQNPs showed a marked decrease of positive cells (Figure 6D). SV and SeNPs reduced the number of positive cells that reacted with the caspase 3 stain (Figure 6E). After direct immunohistochemical staining, the cross-section of the quadriceps muscle of animals treated with SV, coQNPs, and SeNPs showed a marked change in the number of positive cells that react with caspase 3, indicated by dark brown discoloration (Figure 6F). Immunohistochemical examination for apoptosis by determining caspase-3 reactivity revealed a marked positive interaction due to the existence of caspase-3 all over hepatocytes and myocytes in the case of hyperlipidemic rat tissues. A mountain of evidence speculated on the elevation of MDA is associated with a higher cleaved of caspase-3 and apoptotic cells.^70^ Immunohistochemical studies of liver and quadriceps muscles affirmed the apoptotic effects of SV that were indicated by a positive reaction with caspase 3. These results agreed with earlier studies that speculated on the role of SV by downregulating the PI3K/Akt pathway, Akt/protein kinase B (PKB), which controls cell survival and proliferation.^71^

 The research outcomes of the current study showed that combined treatment of SV with coQNPs and SeNPs exhibited a robust anti-hyperlipidemic and antihyperglycemic activity through a significant reduction in TC, TG, LDL-c, and AIX in addition to an increment in HDL-c. At the same time, serum glucose and insulin were normalized. A significant increase in ALT, and AST was detected relative to the SV group, with no significant alterations in ALP and albumin found between SV and this combined therapy. This hepatic effect was confirmed by histopathological examination. Compared to the SV-treated group, an intense caspase-3 expression was found in the rat’s quadriceps muscle.

 Very few studies determined this triple combination’s possible therapeutic efficacy and side effects. Bogsrud et al^72^ reported no significant changes in combined treatment with atorvastatin, CoQ10, and selenium on SIM compared with the placebo. In contrast, our results revealed a marked increase in CK, MYO, and Tn-T compared to treatment with SV alone. Histological and immunohistochemical findings evidenced this unpreferable effect. Histopathological findings in rat’s quadriceps showed muscular atrophy in a focal manner with focal deposition of the fat droplets in between the bundles. The number and intensity of apoptotic cells increased in quadriceps skeletal muscles compared to the SV-treated group. In contrast, no significant difference was determined between combination therapy of SV and coQNPs and SeNPs and treatment with SV in kidney function biomarkers such as creatinine, urea, and BUN. On the other hand, concurrent administration of SV, coQNPs, and SeNPs reduced MDA content with increased GSH content and normalized SOD activity. Results suggest that this triple combination suffers from specific interactions that led to pro-oxidation, which worsens the side effects of SV and interaction circumstances. Immunohistological changes were scored in Table 7.

**Table 7:** Effect of different treatments on immunopathological reaction of caspase-3 in hepatic tissues and quadriceps muscles

**Tissue/Group number**	**Nano-vehicle**	**HFD**	**HFD+SV**	**HFD+SV+coQNPs**	**HFD+SV+SeNPs**	**HFD+SV+coQNPs+SeNPs**
Hepatic tissues	-ve	+ ve	+ ve	-ve	-ve	+ ve
Quadriceps muscle	-ve	+ ve	-ve	-ve	-ve	+ ve

*Note:* + ve, immunoreaction with caspase 3; -ve, No immunoreaction.

## Conclusion

 In summary, this study presents a novel approach utilizing SLNs for the oral delivery of CoQ10 and/or selenium in combination with SV. The findings of this research shed light on the potential applications of coQNPs and SeNPs as therapeutic agents for hyperlipidemia and as a means to mitigate SV-induced side effects.

 The results indicate that the combination therapy of SV with coQNPs and the combined treatment of SV and SeNPs demonstrated improved therapeutic effects compared to SV alone, with reduced side effects. This suggests that the utilization of SLNs can enhance the efficacy of SV treatment in managing hyperlipidemia.

 However, it is essential to note that the simultaneous administration of SV with coQNPs and SeNPs resulted in a higher incidence of hepatotoxicity, nephrotoxicity, and rhabdomyolysis compared to SV alone, despite the observed anti-hyperlipidemic and antihyperglycemic effects. Further research is warranted to optimize these nanoparticles’ dosage and administration protocols to minimize adverse effects while maximizing their therapeutic benefits.

 In the future, it would be beneficial for other researchers to explore alternative formulations and delivery strategies to enhance the bioavailability and effectiveness of CoQ10 and selenium. Additionally, investigations into the underlying mechanisms responsible for the observed hepatotoxicity, nephrotoxicity, and rhabdomyolysis in combination therapy could provide valuable insights for the development of safer treatment regimens.

 Overall, this study lays the groundwork for further exploration of CoQ10 and selenium nanoparticles in the context of hyperlipidemia treatment and offers valuable directions for future research in this field.

## Acknowledgments

 The authors thank Dr. Adel Bekier for his generous support in the histopathological examination in this manuscript.

## Competing Interests

 All authors declare that they have no competing interests.

## Ethical Approval

 This study was conducted with the approval of the Faculty of Pharmacy, Cairo University ethics committee under permit number PT 1353.
